# Antifeedant and ovicidal activities of ginsenosides against Asian corn borer, *Ostrinia furnacalis* (Guenee)

**DOI:** 10.1371/journal.pone.0211905

**Published:** 2019-02-15

**Authors:** Shuangli Liu, Xiaohui Wang, Yonghua Xu, Rui Zhang, Shengyuan Xiao, Yingping Wang, Lianxue Zhang

**Affiliations:** 1 National & Local Joint Engineering Research Center for Ginseng Breeding and Application, Jilin Agricultural University, Changchun, Jilin, China; 2 Research Center of Agricultural Environment and Resources, Jilin Academy of Agricultural Sciences, Changchun, Jilin, China; Macau University of Science and Technology, MACAO

## Abstract

**Introduction:**

Ginsenosides, including protopanaxdiol (PPD) and protopanaxtriol (PPT) type ginsenosides, have been identified as natural insecticidess. This study aimed to investigate the antifeedant and ovicidal activities of total ginsenosides, protopanaxdiol saponins (PDS) and protopanaxtriol saponins (PTS) against Asian corn borer, *O*. *furnacalis* (Guenee).

**Methods and results:**

*O*. *furnacalis* egg masses (> 40 eggs) at 0-, 1- and 2-day-old were dipped into ginsenosides and egg hatchability was significantly inhibited by total ginsenosides, PDS, and PTS in dose and egg-age dependent manners. 100 mg/ml PDS had the strongest ovicidal activity against 0- (80.58 ± 0.95%), 1- (71.48 ± 5.70%), and 2-day-old eggs (64.31 ± 3.20%). In no-choice and choice feeding tests, we observed that the 3^rd^ instar larvae consumed decreased area of leaves treated with ginsenosides, and the antifeedant activities of total ginsenosides, PDS, and PTS against the 3^rd^ instar larvae were time and dose-dependent, with higher activities at 48 h. 100 mg/ml PDS had relative higher antifeedant activity (88.39 ± 3.43% in no-choice and 80.9±4.36% in choice) than total ginsenosides and PTS at all time intervals, except at 48 h in no-choice test. In further experiments, we found PPD ginsenosides (Rb1, Rb2, Rc, and Rd) had relative higher time and dose dependent antifeedant activities than PPT ginsenosides (Re and Rg1).

**Conclusions:**

Our results suggested the insecticidal action of total ginsenosides, PDS, and PTS on *O*. *furnacalis*. All ginsenosides, especially PDS, showed antifeedant and ovicidal activities against *O*. *furnacalis*.

## Introduction

Ginseng contains about 60 species of triterpene saponins [1]. Saponins of ginseng, or ginsenosides, are secondary metabolites mainly synthetized in *Panax ginseng* (*P*. *ginseng*) [2]. Ginsenosides own various pharmacological properties including neuroprotective [3], anti-inflammatory [4, 5], anti-fatigue [6], antiaging [7], antiallergic [6, 8] and antitumor effects [9–12]. In addition, ginsenosides have been reported to be efficient against pest infestation by by preventing oviposition, feeding behavior and egg hatchability [1, 2, 13, 14].

**Fig 1:**
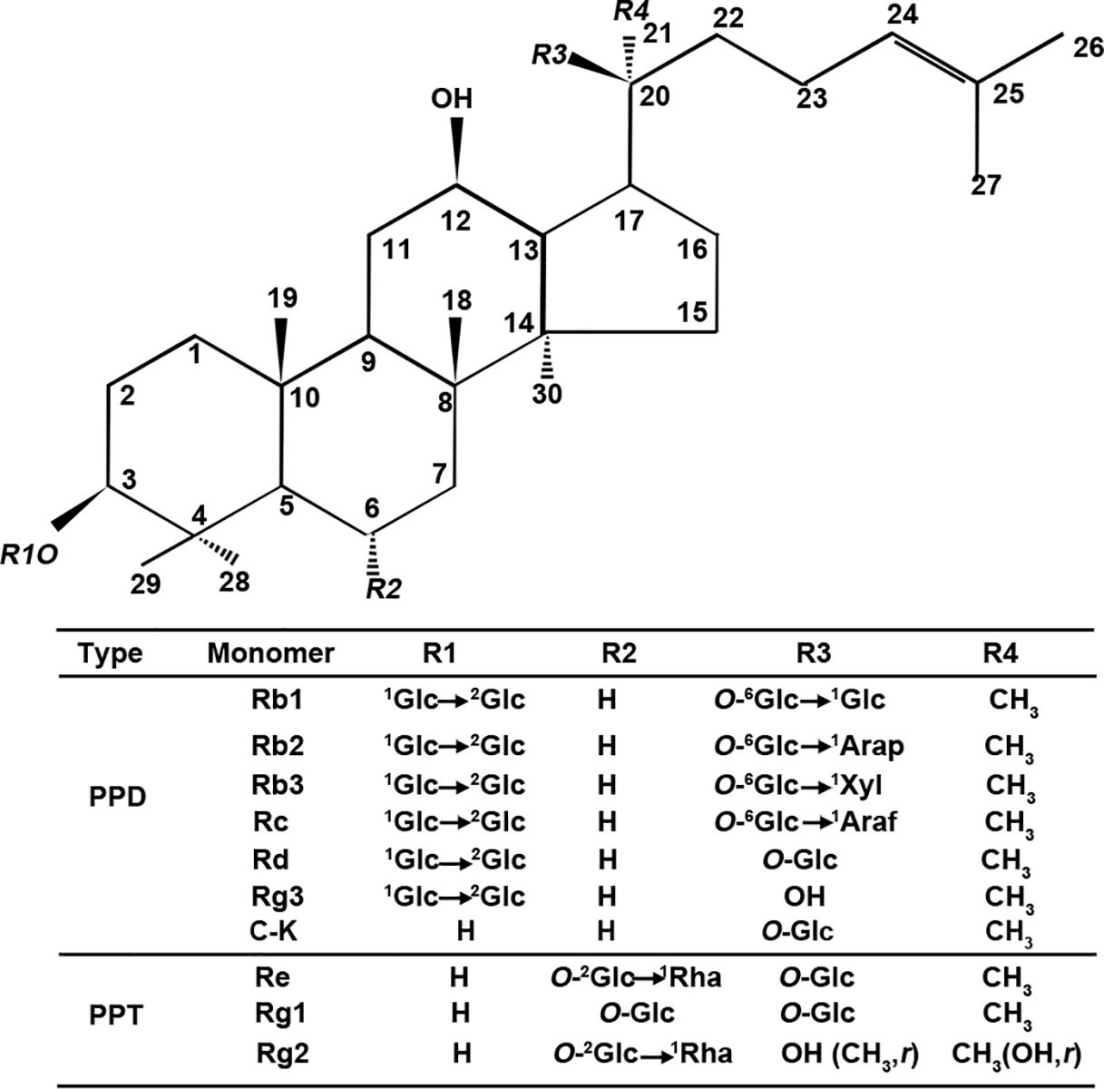
The chemical structure of PPD and PPT type saponins. Glc, β-D-glucopyranosyl; Rha, α-L-rhamnopyranosyl; Ara(f), Arabinose (furanose form); Ara(p), arabinose (pyranose form).

Ginsenosides Rb1, Rb2, Rc, Rd, Re and Rg1 are common compounds in *P*. *ginseng* ginsenosides, accounting for over 80 percentage of total ginsenosides [15]. Rb1, Rb2, Rc, Rd, Rh2 and Rg3 are protopanaxdiol (PPD) type ginsenosides, and Re, Rg1 and Rg2 are protopanaxtriol (PPT) type ginsenosides (Fig 1) [15, 16]. The two type ginsenosides share a common dammarane triterpenoid structure [15]. PPD ginsenosides own two sugar moieties which attach to the β-OH hydroxyl at the C-3 and C-20 positions of PPD. PPT ginsenosides own two sugar moieties which link to the α-OH hydroxyl at C-6 position or β-OH hydroxyl at C-20 position [15, 17]. Previous reports had shown that both panaxadiol saponins (PDS) and panaxatriols saponins (PTS) had low activity [17]. In comparison with PTS (Rg1, Rg2, Re and Rf), PDS (Rg3, Rd, Rc, Rb1, Rb2 and Rh2) shows similar but relative more active roles in anti-inflammatory, neuroprotective, anti-tumor, and anti-fatigue effect [15, 18–20]. However, some studies showed that PTS had more powerful effect on inhibiting cancer cell invasion than PDS [12]. The effects of different monomers are affected by various conditions, such as temperature or structure [18].

Our previous study showed that total ginsenosides from the stems and leaves (GSLS) of *P*. *ginseng* showed significant antifeedant activity against *Plutella xylostella* (Linnaeus) [21]. We observed that the administration of GSLS extracted from *P*. *ginseng* significantly reducted the activity of acetylcholine esterase (AChE), carboxylesterase (CarE) and glutathione S-transferase (GST), while increased mixed-functional oxidase (MFO) activity in *P*. *xylostella*, suggesting the decreasing detoxication capacity in *P*. *xylostella*. Other studies also showed the obvious efficient of ginsenosides against pest infestation by influencing feeding behavior of *Mythimna separata* (*M*. *separata* Walker) Larvae [22]and *Acyrthosiphon pisum* (*A*. *pisum* pea aphid)[14], as well as digestive enzymes in *M*. *separata* larvae [23] and *A*. *pisum* [14]. However, there is less information on the activity of pest ginsenosides against infestation from *Ostrinia furnacalis* (*O*. *furnacalis)*.

*O*. *furnacalis* is a widely distributed insect serviously threatening the production of maize in Asia [24, 25]. The control of *O*. *furnacalis* is very difficult work for agricultural management in Aisa. This study was designed to investigate the antifeedant and ovicidal activities of ginsenosides against *O*. *furnacalis*. The influence of series concentrations of ginsenosides were tested on egg hatchability and feeding behavior (in no-choice and choice situations). The antifeedant and ovicidal activities of total ginsenosides from the leaf and stem of *P*. *ginseng*, PDS, and PTS against *O*. *furnacalis* eggs or the 3^rd^ instar larvae were investigated. This study would provide us with new insights into the insecticidal action of different ginsenosides against *O*. *furnacalis*.

## Materials and methods

### Preparation of total ginsenosides extraction

*P*. *ginseng* was grown at ginseng planting base at Fusong, Jilin, China for four years. Prior to the harvest of ginseng, withered leaves and stems were collected, naturally dried, ground into powder, and stored at ambient temperature. Total ginsenosides fromginseng stems and leaves (GSLS) were extracted from the *P*. *ginseng* leaf and stem powder using water-refluxing method [26]. About 1.0 kg powder was dissolved into 1 L distilled water and refluxed for 2 hours. Rplicated for 3 times with 500 ml water for each time, merged the extract and then concentrated to a total volume of 280 g. The water extract was then diluted with water (100 g/L) and chromatographed on D101 macroporous resin using water and 75% ethanol. The 75% ethanoleluents were dried and total ginsenosides with purity > 90% (UV) were obtained. For extraction of PDS, total ginsenosides were dissolved in 10% NaOH and re-extrated using butyl ethanol. PDS was extracted from NaOH fraction using ethanol sedimentation, and PTS was extracted from the butyl ethanol fraction with 2% NaOH solution and ethanol sedimentation, respectively. PDS (including Rb1, Rb2, Rb3, Rc, Rd, Rg3 and Rh2) and PTS (including Re, Rf, Rg1 and Rg2) were obtained by passing through ion-exchange resin/silica gel column using n-butanol-ethyl acetate-water solution (4:1:2) and chloroform-methanol-ethyl acetate-water eluention (2:2:4:1), respectively. Standards of ginsenosides, including Rg1 (cat#201511), Re (cat#201523), Rf (cat#201537), Rb1 (cat#201501), Rg2 (cat#201545), Rc (cat#201536), 20(R)Rh1 (cat#201515), Rb2 (cat#201551), Rb3 (cat#201518), F1 (cat#201549), Rd (cat#201579), Rk3 (cat#201562), F2 (cat#201521), Rh4 (cat#201571), Rg3 (cat#201506), PPT, C-K (cat#201520), Rg5 (cat#201524), Rh2 (cat#201543), PPD (cat#201512), PDS (cat#201519), and PTS (cat#201513) were obtained from National ginseng engineering center of Jilin Agricultural University, Jilin, China. The purity (> 95%) of all agents was identified an Agilent series 1260 HPLC system (Agilent Technologies, Santa Clara, CA, USA). Agents were diluted into distilled water to the series concentrations of PDS, PTS (5, 10, 25, 50 and 100 mg/ml) and ginsenosides (0.25, 0.5, 1.0, 2.0, and 4.0 mg/ml) before experiments.

### Insects

The *O*. *furnacalis* eggs were obtained from the Institute of Biocontrol, Jilin Agricultural University, China, and maintained in an intelligent artificial climate chamber (PRS-20, Ningbo Saifu Laboratory Instrument Factory, Ningbo, China) at 27 ± 1°C with a relative humidity of 70 ± 5% and a light cycle of 14 h day: 10 h night. Insects were fed with artificial diets for 5 generations with undisturbed mating behavior. The indoor populations of *O*. *furnacalis* and their eggs were then subjected to treatments with ginsenosides.

### Ovicidal activity bioassay

For ovicidal bioassay, 10 egg masses (≥ 40 eggs in each egg mass) at each age (0-, 1-, and 2-day-old) of the indoor populations of *O*. *furnacalis* were immersed into solutions with different concentrations of ginsenosides (0, 5, 25, 50 and 100 mg/ml) for 10 s. After eggs masses were air dried, 10 eggs were placed per 9cm petri dish each containing a moistened filter paper, with 10 replications per treatment. Dishes were incubated in a chamber as described above. The unhatched eggs (unbroken eggs) were counted daily using a Leica binocular microscope (Leica Microsystems, Germany) until hatching ceased. Control experiments were conducted by dipping egg masses into distilled water for 10 s. Each experiment was repeated for 10 times. The egg hatchability (eggH) was calculated as: eggH(%) = (hatched eggs)×100/total eggs. The ovicidal activity of ginsenoside was repressed by the adjusted inhibition rate of egg hatchability, which was calculated as: Adjusted inhibition rate of egg hatchability(%) = (eggH_control_—eggH_test_)×100/eggH_control_.

### Feeding bioassay

Leaf disc choice and no-choice methods were used to study the larvicidal activity. Fresh maize leaves were collected from ‘Xianyu 335’ at jointing stage. Leaves were imidiately stored at 4°C before experiments. Leaf discs (15 mm in diameter) were dipped into solutions with different concentrations of ginsenosides (0, 5, 25, 50 and 100 mg/ml) for 10 s. Leaf discs dipped into distilled water were used as negative control. The leaf discs were placed on filter papers and air dried. In choice bioassay, 3 test discs and 3 negative control discs dipped into the same solutions were put into one petri dish (9 cm in diameter) with interval [21]. In no choice bioassay, 6 test discs were put into one dish, and dishes with 6 control discs were set as negative controls [21]. Each experiment was replicated for 10 times. The 3^rd^ instar larvae were fasted for 4 h and then evenly placed onto the empty positions of dishes (n = 6 in each dish) with leaf discs. All dishes were maintained in a chamber as described above for 24 h, 48 h and 72 h. The remaining area (ReA) of individual leaf disc was determined using a leaf area meter (LI-3000; LI-COR, Lincoln, NE, USA). Then, the antifeedant activity of ginsenoside against *O*. *furnacalis* larvae for choice bioassay was computed as: antifeedant activity(%) = (ReA_control_—ReA_test_)×100/(ReA_control_ + ReA_test_), and that for no-choice assay was computed as: antifeedant activity(%) = (ReA_control_—ReA_test_)×100/(ReA_control_).

It has been reported that Rb1, Rb2, Rc, Re and Rg1 are major compositions (80%) of total ginsenosides [15]. As we described above, Re, Rd, Rg1, Rb3 and Rb1 are major compositions (~ 45%) of GSLS from *P*. *ginseng*; PDS was mainly composted of PPD type ginsenosides including Rd, Rc, Rb2, Rb1 and Rb3 [15, 17]; and PTS was essentially consisted of Re, Rg1, and Rg2 (accounting for ~ 55%) [17]. To investigate the reasons causing the difference in antifeedant activity among GSLS, PDS, and PTS, we fed the 3^rd^ instar larvae with leaf discs treated with different active ginsenoside monomers, including PPD ginsenosides (Rb1, Rb2, Rc, and Rd) and PPT type ginsenosides (Re and Rg1). Experiments were performed as aforementioned for 10 replications in each condition.

### Statistical analysis

All data were expressed as mean ± SD from 10 replications. Statistical analyses were performed using SPASS 22.0 software. Data were analyzed using one-way ANOVA followed by a post hoc Dunnet’s test for comparion. Median lethal concentration (LC_50_) and antifeedant concentration (AFC_50_) of ginsenosides were calculated using regression analysis. P < 0.05 was set as significant different.

## Results and discussion

### HPLC analysis of ginsenosides composition

**Fig 2:**
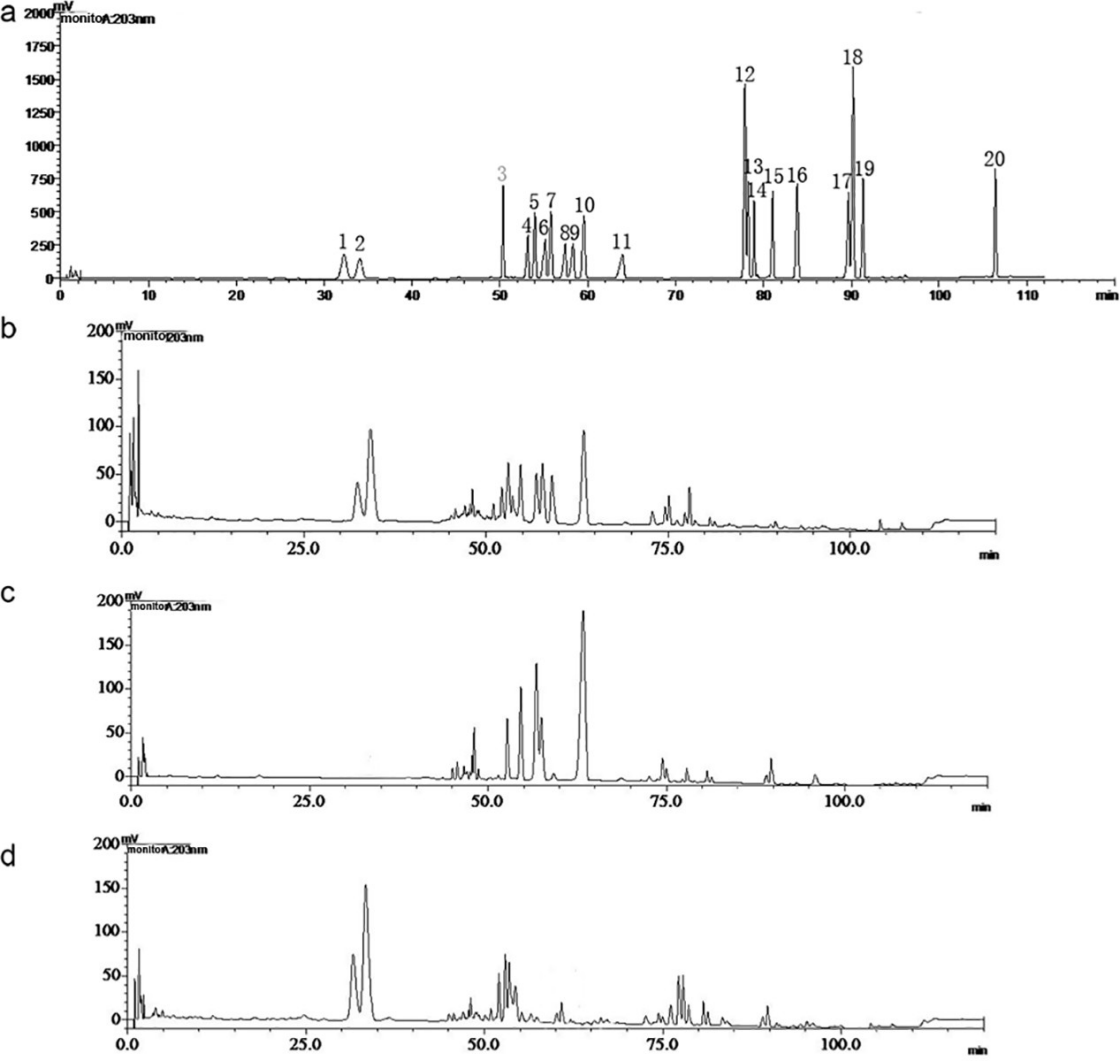
Composition of ginsenosides by HPLC. a, chromatogram of the 20 standard ginsenosides by HPLC analysis. Number 1–20 indicates composition of Rg1, Re, Rb1, Rg2, Rc, Rb2, Rb3, F1, Rd, F2, Rh4, Rg3, protopanaxatriol (PPT), compound K (CK), Rh1, Rf, and protopanaxadiol (PPD), respectively. Chromatogram of total ginsenosides from stem and leaf of *Panax ginseng* (b, GSLS), panaxadiols saponins (c, PDS) and panaxatriol saponins (d, PTS).

Qualitative and quantitative analysis of GSLS, PDS, and PTS is shown in Fig 2 and Table 1. GSLS from the leaf and stem of *P*. *ginseng* is characterized by the presence of 14 compositions, including Rg1, Re, Rb1, Rg2, Rc, Rb2, Rb3, F1, Rd, F2, Rh4, Rg3, C-K, and PPD (Fig 2A and 2B). PDS is characterized by the presence of Rb1, Rb2, Rb3, Rc, Rd, Rg3 and Rh2 (Fig 2C and Table 1), and PTS is characterized by Rg1, Re, and Rg2 (Fig 2D and Table 1), respectively. The major compositions of GSLS from the leaf and stem of *P*. *ginseng* are Re (15.59 ± 0.95%), Rd (12.73 ± 0.21%), Rg1 (5.80 ± 0.82%), Rb3 (5.68 ± 0.18%) and Rb1 (5.18 ± 0.07%, Table 1). The major compositions of PDS are Rd (27.8 ± 0.21%), Rb2 (15.81 ± 0.16%) and Rc (9.67 ± 0.29%). PTS is mainly characterized by Re (33.87 ± 0.17%), Rg1 (14.15 ± 0.29%), and Rg2 (7.57 ± 0.16%, Table 1).

**Table 1:** Composition content of total ginsenoside from the stem and leaves of *Panax ginseng*, and PDS and PTS.

No.	Standards	GSLS (%)	PDS (%)	PTS (%)
1	Rg1	**5.80 ± 0.82**	-	**14.15 ± 0.29**
2	Re	**15.59 ± 0.95**	-	**33.87 ± 0.17**
3	Rf	-	-	1.56 ± 0.03
4	Rb1	5.18 ± 0.07	5.45 ± 0.07	-
5	Rg2	1.38 ± 0.15	-	7.57 ± 0.16
6	Rc	4.88 ± 0.29	9.67 ± 0.29	-
7	20(R)Rh1	-	-	-
8	Rb2	4.83 ± 0.16	**15.81 ± 0.16**	-
9	Rb3	**5.68 ± 0.18**	8.21 ± 0.18	-
10	F1	3.04 ± 0.17	-	-
11	Rd	**12.73 ± 0.21**	**27.8 ± 0.21**	-
12	Rk3	-	-	-
13	F2	1.34 ± 0.08	-	-
14	Rh4	0.52 ± 0.02	-	-
15	Rg3	0.15 ± 0.01	0.41 ± 0.001	-
16	PPT	-	-	-
17	C-K	0.05 ± 0.01	-	-
18	Rg5	-	-	-
19	Rh2	-	6.79 ± 0.28	-
20	PPD	0.14 ± 0.01	-	-
Total		61.23 ± 2.08	74.14 ± 1.21	57.15 ± 1.06

GSLS, total ginsenosides; PDS, panaxadiols saponins; PTS, panaxatriol saponins.

Our analysis results suggested that there were differences in compositions of GSLS, PDS and PTS, which might suggest the different activities of GSLS, PDS and PTS against *O*. *furnacalis*.

### Ovicidal activity of GSLS, PDS and PTS against the corn borer eggs

**Fig 3:**
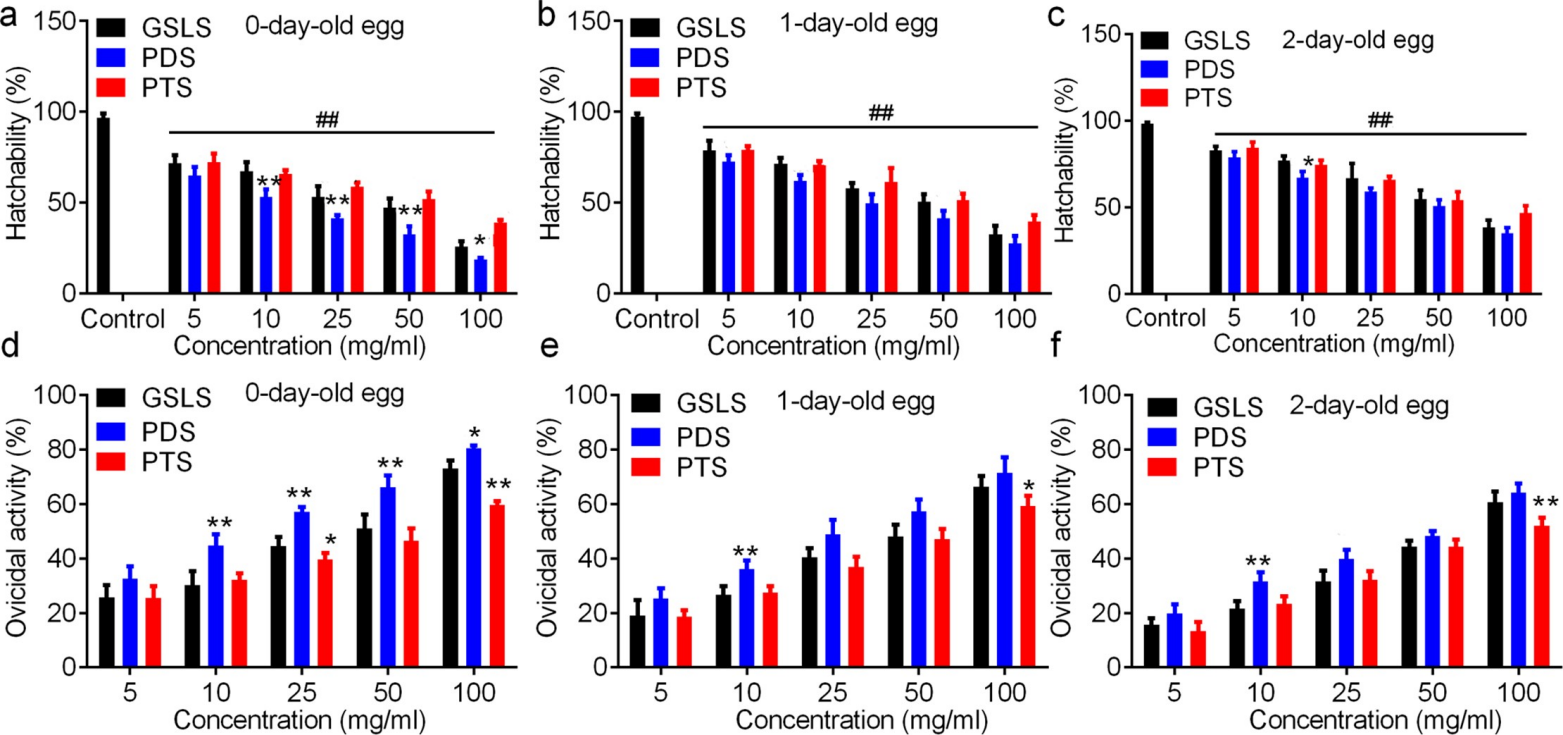
Hatchability and ovicidal activity of ginsenosides against *O*. *furnacalis* eggs. a-c, hatchability of *O*. *furnacalis* eggs treated with ginsenosides. d-f, ovicidal activity of GSLS, PDS, and PTS against *O*. *furnacalis*eggs at 0-day-old, 1-day-old and 2-day-old, respectively. * and ** notes p < 0.05 and 0.01 vs. total ginsenosides (GSLS) in each concentration, respectively. ## indicates p < 0.01 vs. control in Fig 3A–3C. Original data is listed in S1 Table. GSLS, total ginsenosides from of ginseng stems and leaves; PDS, panaxadiols saponins; PTS, panaxatriol saponins.

We firstly analyzed the inhibitory effect of GSLS, PDS and PTS against the hatchability of *O*. *furnacalis* eggs. Fig 3 shows that all ginsenosides significantly inhibited egg hatchability in a dose-dependent manner. The detail data were listed in S1 Table. After being treated with GSLS (5 ~ 100 mg/ml), the hatchability of eggs at 0-, 1- and 2-day-old was in a range of 71.70% ~ 25.98%, 78.67% ~ 32.69% and 38.61% ~ 82.86%, respectively, which was lower than 96.86% ~ 98.38% of control (Fig 3A–3C and S1 Table). The hatchability of 0-day-old eggs treated by PDS was significantly lower than eggs treated by GSLS and PTS at the same concentration, while that of the 1- and 2-day-old eggs treated by PDS was insignificantly lower than GSLS and PTS (Fig 3A–3C).

On contrast, the ovicidal activity of ginsenosides was gradually increased with ginsenoside concentrations (S1 Table and Fig 3D–3F). The ovicidal activity of 100 mg/ml PDS was the highest (80.58±0.95%, 71.48±5.70% and 64.31±3.20%) against *O*. *furnacalis* eggs (at 0-, 1- and 2-day-old). We found the ovicidal activity of PDS for 0-, 1-, and 2-day-old eggs was higher than that of GSLS and PTS in each concentration (Fig 3D–3F). These results were consistent with our previous report showing the activity of GSLS against oviposition of *Pieris rapae* [2]. Our results confirmed the ovicidal activity of GSLS, PDS and PTS against the hatch of *O*. *furnacalis* eggs, suggesting that GSLS, PDS and PTS were potential inhibitors of *O*. *furnacalis* egg hatch.

Regression analysis showed that PDS exhibited the LC_50_ values of 23.01 mg/ml, 41.20 mg/ml, and 59.75 mg/ml for ovicidal activity against eggs at 0-, 1-, and 2-day-old, respectively, which was lower than LC_50_ of GSLS (48.08 mg/ml, 58.78 mg/ml and 71.63 mg/ml) and LC_50_ of PTS (65.56 mg/ml, 67.29 mg/ml, and 73.99 mg/ml; Table 2) based on confidence intervals. These results suggested that GSLS, PDS and PTS all inhibited the *O*. *furnacalis* egg hatchability in dose-dependent manners, and egg age affected the ovicidal activity of ginsenosides. PDS had relative higher ovicidal activity against *O*. *furnacalis* eggs at all ages (0 ~ 2-day-old) compared with PTS and GSLS from the leaf and stem of *P*. *ginseng*, based on LC_50_ values and non-overlapping confidence intervals.

**Table 2:** Regression analysis for ovicidal activity of ginsenosides.

Types	LC_50_ (mg/ml)	R^2^	95% CI
**0-day-old**			
GSLS	48.08	0.9698	46.70 ~ 49.46
PDS	**23.01**	0.9163	21.03 ~ 24.99
PTS	65.56	0.9373	63.32 ~ 67.99
**1-day-old**			
GSLS	58.78	0.9258	56.35 ~ 61.22
PDS	**41.20**	0.8968	38.73 ~ 43.66
PTS	67.91	0.8891	64.63 ~ 71.19
**2-day-old**			
GSLS	71.63	0.9443	69.29~73.97
PDS	**59.75**	0.9080	56.08~ 62.60
PTS	73.99	0.8948	70.61 ~ 77.36

GSLS, total ginsenosides; PDS, panaxadiols saponins; PTS, panaxatriol saponins. LC50: median lethal concentration.

### Antifeedant activity of GSLS, PDS and PTS against the 3^rd^ instar corn borer larvae

**Fig 4:**
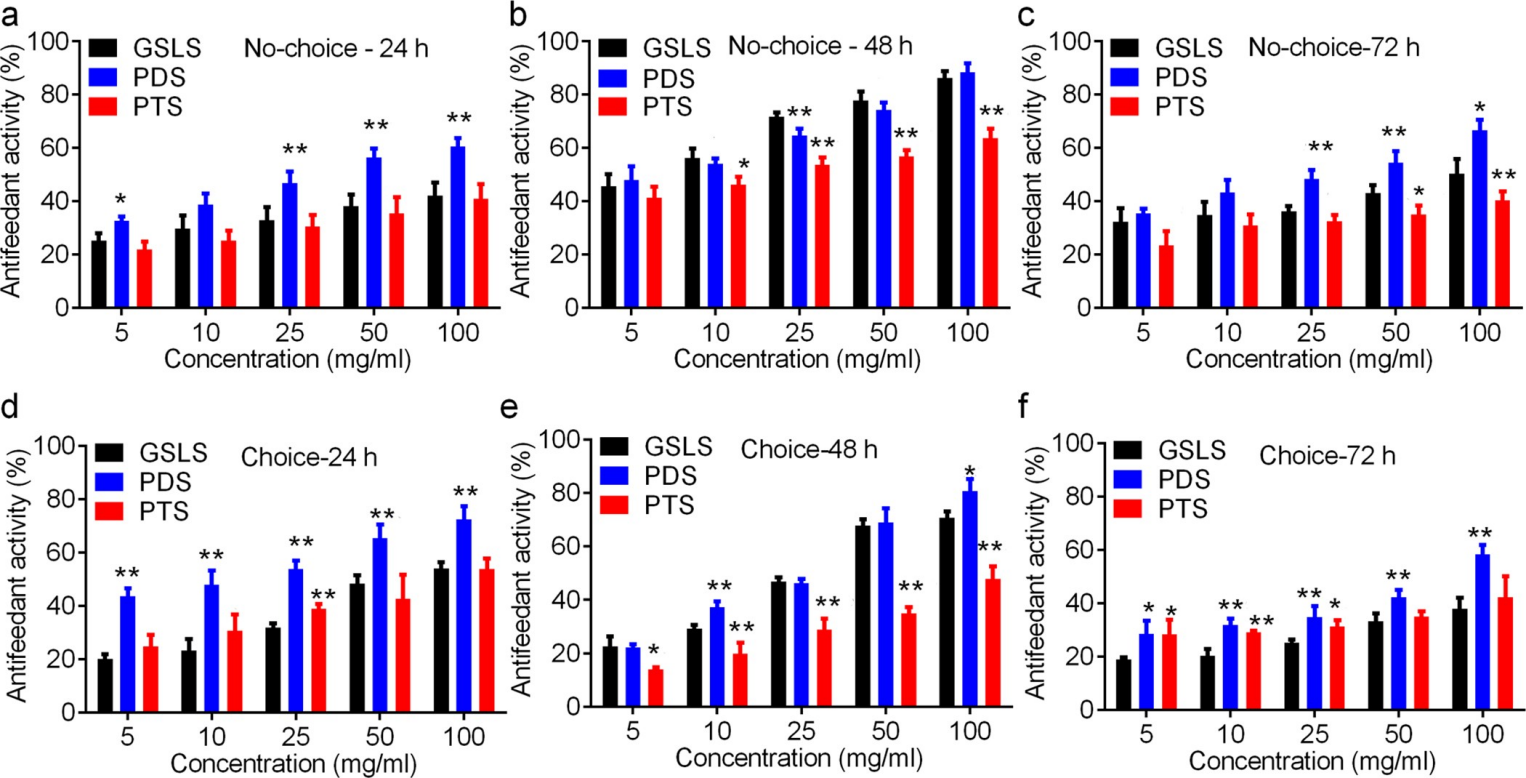
Antifeedant activity of ginsenosides against the 3^rd^ instar larvae of *O*. *furnacalis*. a-c, no-choice antifeedant activity of ginsenosides against *O*. *furnacalis* larvae for 24 h, 48, and 72 h, respectively. d-f, choice antifeedant activity of ginsenosides against *O*. *furnacalis* larvae for 24 h, 48, and 72 h, respectively. * and ** notes p <0.05 and 0.01 vs. GSLS (total ginsenosides from of ginseng stems and leaves) in each concentration, respectively. PDS, panaxadiols saponins; PTS, panaxatriol saponins. Original data of no-choice and choice antifeedant activity analysis are available from S2 and S3 Tables, respectively.

Fig 4 shows the no-choice and choice antifeedant activities of GSLS, PDS and PTS against the 3^rd^ instar larvae of *O*. *furnacalis* were time and dose-dependent. The consumed leaf area (mm^2^) was gradually decreased with increased ginsenoside concentrations (S2 and S3 Tables). Larvae that fed on the leaf discs treated with highest ginsenoside concentrations (100 mg/ml) consumed the minimum leaf area, suggesting the highest antifeedant activity of 100 mg/ml ginsenosides against the 3^rd^ instar larvae (Fig 4 and S2 and S3 Tables). In addition, PDS showed higher antifeedant activity than others in the same concentration (S2 and S3 Tables).

In no-choice assay, GSLS, PDS and PTS showed relative higher antifeedant activity against instar larvae at 48 h than those at 24 h and 72 h. At 48 h, 100 mg/ml GSLS, PDS and PTS exhibited 86.37 ± 2.38%, 88.39 ± 3.43%, and 63.76 ± 3.56% antifeedant activity against the 3^rd^ instar larvae of *O*. *furnacalis*, respectively. In addition, we found PDS exhibited the highest antifeedant activity against the 3^rd^ instar larvae at all concentrations after 24 h and 72 h, while GSLS showed higher antifeedant activity at 48h than PDS and PTS in all concentration (Fig 4A–4C and S2 Table).

Choice bioassay revealed PDS had the highest antifeedant activity against the 3^rd^ instar larvae in all experiments (Fig 4D–4F). 100 mg/ml PDS showed the highest antifeedant activity of 70.88 ± 2.34%, 80.9 ± 4.36%, and 47.88 ± 4.71% at 24 h, 48 h, and 72 h, respectively, and those were significantly higher than GSLS and PTS (p <0.05, S3 Table). In no-choice and choice assays, antifeedant activities of ginsenosides were relative lower at 72 h than those at 24 h and 48 h. These results of no-choice and choice bioassays agreed with our previous studies showing the inhibitory activity of GSLS against insect feeding behavior [1, 2, 21]. Previous studies had determined the antifeedant activity of GSLS against the 3^rd^ instar larvae of *Pieris rapae* [2], the 4^th^ instar larvae of *M*. *separata* [1] and *Plutella xylostella* (Linnaeus) [21].

We calculated the choice and no-choice AFC_50_ of GSLS, PDS and PTS at all time intervals. Regression analysis of no-choice bioassay showed PDS exhibited the lowest AFC_50_ value of 30.86 mg/ml, 7.06 mg/ml, and 23.61 mg/ml at 24 h, 48 h and 72 h, respectively (Table 3). AFC_50_ values of PTS in choice and no-choice tests were higher than those of GSLS and PDS based on LC_50_ values.

**Table 3:** Regression analysis for antifeedant activity of ginsenosides.

Types	No-choice		Choice	
	AFC_50_(mg/ml)	R^2^	AFC_50_ (mg/ml)	R^2^
**24 h**				
GSLS	**269.65**	0.9881	70.19	0.9466
PDS	30.86	0.9900	26.09	0.9632
PTS	457.09	0.9920	276.93	0.9778
**48 h**				
GSLS	6.34	0.9882	11.85	0.9626
PDS	7.06	0.9776	21.57	0.9750
PTS	16.44	0.9927	62.77	0.8457
**72 h**				
GSLS	118.87	0.8921	79.59	0.9744
PDS	23.61	0.9599	149.80	0.9752
PTS	633.98	0.9303	153.05	0.9998

GSLS, total ginsenosides; PDS, panaxadiols saponins; PTS, panaxatriol saponins. AFC50: median antifeedant concentration.

Taken together, these results revealed that GSLS, PDS and PTS all showed antifeedant activities against the 3^rd^ instar larvae of *O*. *furnacalis*, and PDS had the highest antifeedant activity. Fresh maize leaf treated with higher ginsenoside concentrations exhibited higher antifeedant activity against the 3rd instar larvae of *O*. *furnacalis*, suggesting the potential strategies of using ginsenosides for *O*. *furnacalis* management.

### Antifeedant activity of different ginsenoside monomers against the 3^rd^ instar larvae of *O*. *furnacalis*

**Fig 5:**
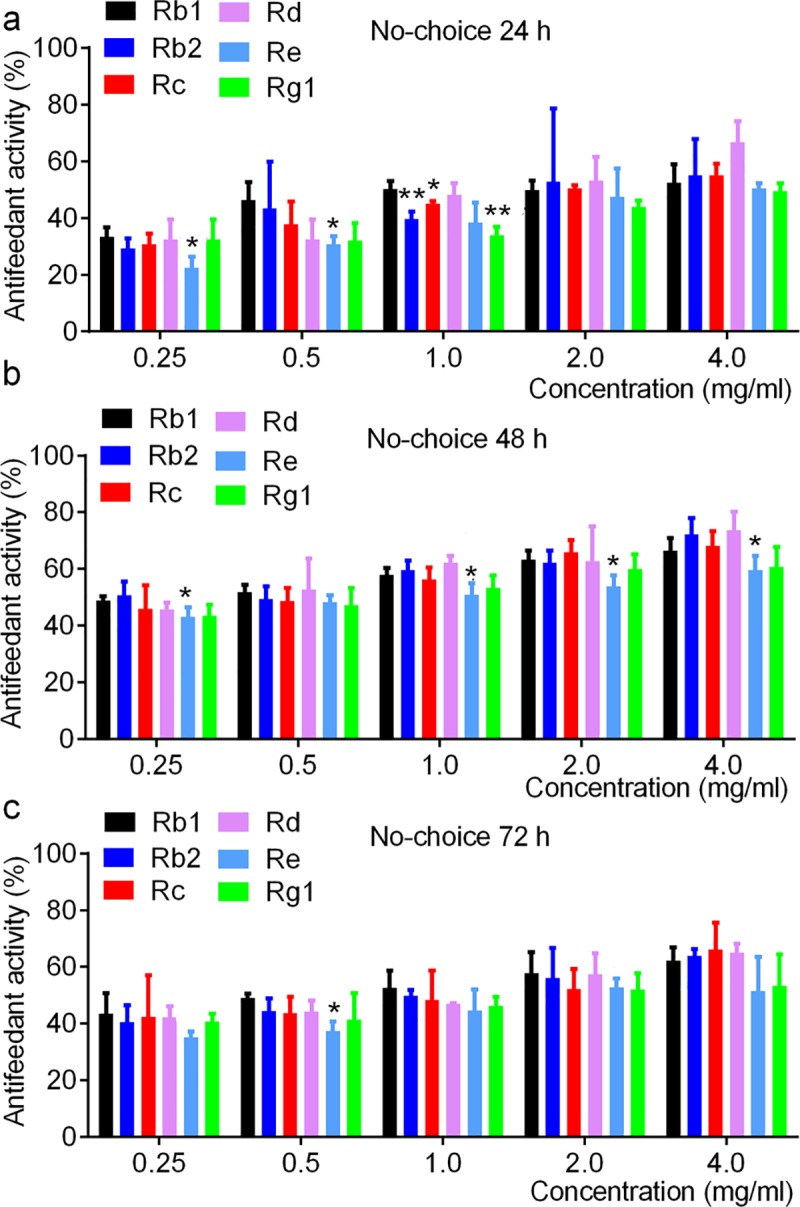
No-choice antifeedant activity of different ginsenoside monomers against the 3^rd^ instar larvae *O*. *furnacalis*. a-c, antifeedant activity of different ginsenoside monomers against the 3^rd^ instar larvae of *O*. *furnacalis* at 24, 48, and 72 h, respectively. * and ** notes p <0.05 and 0.01 vs. Rb1 in each concentration, respectively. Original data are available from S4 Table.

**Fig 6:**
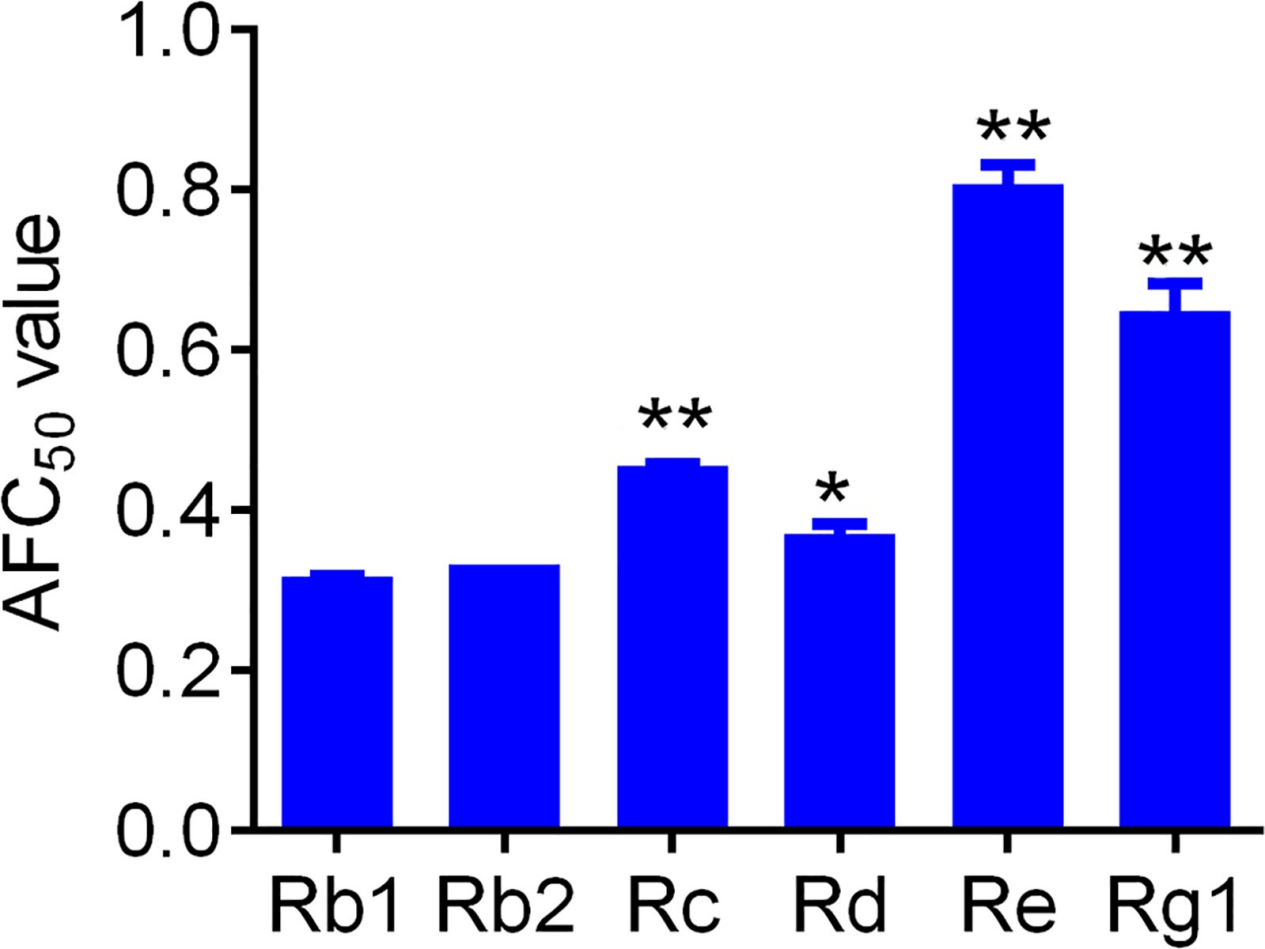
The Median antifeedant concentration (AFC_50_) of ginsenosides at 48 h. * and ** notes p < 0.05 and 0.01 vs. Rb1, respectively.

Fig 5 shows the no-choice antifeedant activities of different ginsenoside monomers against the 3^rd^ instar larvae of *O*. *furnacalis* were gradually increased with the concentrations. The changes of gradually increased antifeedant activities of different ginsenoside monomers were basically similar with small differences and in a dose and time dependent way (S4 Table). All ginsenoside monomers showed relative higher antifeedant activity against *O*. *furnacalis* larvae at 48 h, followed by 72 h and then 24 h (Fig 5 and S4 Table). In comparison among monomers, we found Re and Rg1 monomers in the same concentrations showed relative lower antifeedant activities than Rb1, Rb2, Rc and Rd, and the antifeedant activities of Rb1, Rb2, Rc and Rd were at the same level (Fig 5 and S4 Table). Regression analysis showed the AFC_50_ value of Rb1, Rb2, Rc, Rd, Re and Rg1 was 0.32 mg/ml, 0.33 mg/ml, 0.45 mg/ml, 0.37 mg/ml, 0.81 mg/ml and 0.64 mg/ml at 48 h, respectively (Fig 6).

Previous reports had shown that compound K and Rg3 (PPD type) have various pharmacological activities, including anti-tumorigenesis, anti-inflammatory, neuroprotective and anti-aging [6, 27–29]. Ginsenosides Rb1, Rb2, Re and Rd could be converted into pharmacologically active components PPD type ginsenosides compound K and Rg3 during biotransformation [28, 29]. Oh et al reported that PPT and PPD showed comparable anxiolytic effect on rats and the latter showed stronger anti-inflammatory effect than the former [20]. These studies suggested that the diversity biological in activities of ginsenoside are dependent on the structures and biotransformation pathways. These might explain the differences in the ovicidal and antifeedant activities among GSLS, PDS and PTS ginsenosides against *O*. *furnacalis*.

## Conclusion

In conclusion, our present study determined the insecticidal action of GSLS, PDS and PTS against *O*. *furnacalis* in laboratory conditions. We confirmed that the ovicidal activities of ginsenosides GSLS, PDS and PTS against *O*. *furnacalis* eggs were dose and egg-age dependent. PDS showed the highest ovicidal activity among the three types (GSLS, PDS, and PTS) against *O*. *furnacalis* eggs at 0 ~ 2-day-old. No-choice and choice antifeeding tests showed the time- and dose-dependent antifeedant activities of GSLS, PDS and PTS against the 3^rd^ instar larvae of *O*. *furnacalis*. Moreover, PDS exhibited relative stronger activity against feeding behavior of the 3^rd^ instar larvae than PTS and GSLS. The minor but interesting differences in antifeedant activities among ginsenoside monomers (PPD type ginsenosides Rb1, Rb2, Rc, and Rd; and PPT type ginsenosides Re and Rg1) showed the ginsenoside structures might be associated with the insecticidal activities of ginsenosides. Accordingly, we confirmed the potential of using ginsenosides, especially PDS, as the treatment strategies for controlling *O*. *furnacalis*. The potential should be validated in field experiments. Moreover, the uncovering of molecular mechanism in insects in response to different ginsenosides might be helpful for understanding the insecticidal action of GSLS, PDS and PTS against *O*. *furnacalis*.

## Supporting information

S1 Table The inhibitory rate of total ginsenosides on hatchability of *O*. *furnacalis* eggs at different ages. (DOCX)

S2 Table No-choice antifeedant activity of ginsenosides against the 3^rd^-instar larvae of *O*. *furnacalis*. (DOCX)

S3 Table Choice antifeedant activity of ginsenosides against 3^rd^-instar larvae. (DOCX)

S4 Table No-choice antifeedant activity of different ginsenoside monomers against the 3^rd^-instar larvae of *O*. *furnacalis*. (DOCX)
