# Supplementary material for: Antifeedant and ovicidal activities of ginsenosides against Asian corn borer, Ostrinia furnacalis (Guenee)
Source: PLoS One. 2019 Feb 15;14(2):e0211905. doi: 10.1371/journal.pone.0211905 (PMC6377112; doi:10.1371/journal.pone.0211905)
Supplement: S1 Table — (DOCX) [file pone.0211905.s001.docx]

Supplementary Table S1. The inhibitory rate of total ginsenosides on hatchability of *O. furnacalis* eggs at different ages.

| Con. (mg/ml) | 0-day-old | | 1-day-old | | 2-day-old | |
| --- | --- | --- | --- | --- | --- | --- |
|  | eggH(%) | Activity (%) | eggH(%) | Activity (%) | eggH(%) | Activity (%) |
| CK | 96.86 ±2.38 |  | 97.27 ±1.84 |  | 98.38±0.75 |  |
| **GSLS** |  |  |  |  |  |  |
| 5 | 71.70±4.35 | 25.70±4.50 d | 78.67±5.39 | 19.13±5.54 e | 82.86±2.33 | 15.76±2.37 e |
| 10 | 67.24±5.02 | 30.21±5.20 d | 71.37±3.27 | 26.63±3.36 d | 77.06±2.70 | 21.62±2.75 d |
| 25 | 53.32±5.81 | 44.59±3.39 c | 57.82±3.04 | 40.56±3.12 c | 66.95±8.35 | 31.71±3.80 c |
| 50 | 47.27±4.88 | 51.02±5.10 b | 50.44±4.22 | 48.15±4.34 b | 54.75±5.23 | 44.31±2.31 b |
| 100 | 25.98±2.89 | 73.07±2.99 a | 32.69±4.79 | 66.45±3.92 a | 38.61±3.84 | 60.73±3.91 a |
| **PDS** |  |  |  |  |  |  |
| 5 | 65.03±4.50 | 32.62±4.61e | 72.59±3.61 | 25.37±3.71d | 78.89±3.33 | 19.75±3.37 e |
| 10 | 53.31±3.94 | 44.76±4.08d | 62.02±3.12 | 36.17±3.2 c | 67.14±3.73 | 31.71±3.15d |
| 25 | 41.38±1.74 | 57.12±1.80c | 49.73±5.04 | 48.88±5.28b | 59.02±2.22 | 39.99±3.26c |
| 50 | 32.72±4.27 | 66.09±4.42b | 41.46±4.21 | 57.38±4.31 b | 50.78±3.61 | 48.35±1.67b |
| 100 | 18.74±0.92 | 80.58±0.95a | 27.55±4.09 | 71.48±5.70a | 35.09±3.15 | 64.31±3.20a |
| **PTS** |  |  |  |  |  |  |
| 5 | 72.45±4.71 | 25.52±4.38e | 79.05±2.27 | 18.73±2.33e | 84.43±3.33 | 13.25±3.42e |
| 10 | 65.82±2.16 | 32.34±2.22d | 70.59±2.45 | 27.43±2.52 d | 74.46±2.59 | 23.45±2.66d |
| 25 | 58.69±2.33 | 39.66±2.39c | 61.42±7.72 | 36.86±3.83c | 65.97±2.12 | 32.18±3.16c |
| 50 | 51.94±4.29 | 46.60±4.41b | 51.47±3.63 | 47.08±3.73 b | 54.20±4.81 | 44.27±2.67b |
| 100 | 39.18±1.36 | 59.72±1.37a | 39.58±3.43 | 59.31±3.64a | 46.71±4.15 | 51.98±3.05a |

Data are expressed as mean ± SD. Data were analyzed using one-way ANOVA followed by a post hoc Dunnet’s test for comparison. eggH, egg hatchability; Ovicidal activity is indicated by adjusted inhibition rate of egg hatchability (%). GSLS, toatal ginsenoside of ginseng stems and leaves; PDS, panaxadiols saponins; PTS, panaxatriol saponins. CK, control.
