# Supplementary material for: Antifeedant and ovicidal activities of ginsenosides against Asian corn borer, Ostrinia furnacalis (Guenee)
Source: PLoS One. 2019 Feb 15;14(2):e0211905. doi: 10.1371/journal.pone.0211905 (PMC6377112; doi:10.1371/journal.pone.0211905)
Supplement: S2 Table — (DOCX) [file pone.0211905.s002.docx]

Supplementary Table S2. No-choice antifeedant activity of ginsenosides against the 3^rd^-instar larvae of *O. furnacalis*.

| Con. (mg/ml) | 24 h | | 48 h | | 72 h | |
| --- | --- | --- | --- | --- | --- | --- |
|  | C.area (mm^2^) | Activity (%) | F. area (mm^2^) | Activity (%) | F. area (mm^2^) | Activity (%) |
| CK | 513.67±46.92 | -- | 916.33±58.59 | -- | 603.67±29.28 |  |
| **GSLS** |  |  |  |  |  |  |
| 5 | 383.43±16.28 | 25.31±2.68d | 455.32±34.86 | 45.67±4.45e | 409.33±33.95 | 32.28±5.19c |
| 10 | 363.46±23.03 | 29.85±4.76cd | 401.47±32.59 | 56.31±3.51d | 393.19±26.67 | 34.96±4.75c |
| 25 | 344.67±27.83 | 32.97±4.88bc | 258.61±21.38 | 71.88±1.47c | 386.27±19.30 | 36.20±2.02c |
| 50 | 316.47±25.89 | 38.16±4.49ab | 203.54±19.49 | 77.89±3.3b | 344.84±22.76 | 43.15±2.86b |
| 100 | 290.05±21.58 | 42.12±5.02a | 125.67±9.18 | 86.37±2.38a | 301.12±18.21 | 50.35±5.42a |
| **PDS** |  |  |  |  |  |  |
| 5 | 345.24±20.07 | 32.76±1.52d | 475.33±33.97 | 47.97±5.11e | 391.84±25.47 | 35.46±1.73d |
| 10 | 314.67±28.36 | 38.82±4.11c | 416.25±26.06 | 54.02±2.11d | 343.63±4.73 | 43.35±4.63bc |
| 25 | 272.15±19.63 | 46.83±4.37b | 332.67±36.12 | 64.76±2.56c | 312.67±19.50 | 48.43±3.35b |
| 50 | 223.27±9.85 | 56.47±3.43a | 234.52±22.07 | 74.38±2.75b | 275.33±26.02 | 54.55±4.35b |
| 100 | 203.29±18.53 | 60.5±3.31a | 105.31±13.45 | 88.39±3.43a | 202.18±17.21 | 66.71±3.9a |
| **PTS** |  |  |  |  |  |  |
| 5 | 401.15±19.66 | 21.89±3.01e | 537.15±44.64 | 41.31±4.21d | 464.27±41.56 | 23.58±5.10c |
| 10 | 382.24±18.68 | 25.26±3.73cd | 487.24±34.77 | 46.31±2.87c | 419.41±33.93 | 30.89±4.14b |
| 25 | 358.23±25.50 | 30.62±4.24bc | 425.35±36.97 | 53.75±2.68b | 409.61±26.86 | 32.43±2.34b |
| 50 | 329.68±18.88 | 35.49±6.07ab | 394.38±24.37 | 56.91±2.25b | 393.60±18.01 | 35.03±3.41b |
| 100 | 303.11±19.55 | 40.96±5.56a | 333.23±21.10 | 63.76±3.56a | 362.29±23.89 | 40.35±3.35a |

Data are expressed as mean ± SD. Letters after the data indicate analysis using one-way ANOVA followed by a post hoc Dunnet’s test for comparison. GSLS, toatal ginsenoside of ginseng stems and leaves; PDS, panaxadiols saponins; PTS, panaxatriol saponins. CK, control. C. area, consumed leaf area calculated as: origin area of leaf discs – remaining area (ReA) of leaf discs. Antifeedant activity for no-choice bioassay was computed as: antifeedant activity (%) = (ReA_control_ - ReA_test_)×100%/(ReA_control_).
