# Supplementary material for: Antifeedant and ovicidal activities of ginsenosides against Asian corn borer, Ostrinia furnacalis (Guenee)
Source: PLoS One. 2019 Feb 15;14(2):e0211905. doi: 10.1371/journal.pone.0211905 (PMC6377112; doi:10.1371/journal.pone.0211905)
Supplement: S3 Table — (DOCX) [file pone.0211905.s003.docx]

Supplementary Table S3. Choice antifeedant activity of ginsenosides against 3^rd^-instar larvae.

| Con. (mg/ml) | 24 h | | | 48 h | | 72 h | |
| --- | --- | --- | --- | --- | --- | --- | --- |
|  | F.area (mm^2^) | | Activity (%) | F. area (mm^2^) | Activity (%) | F. area (mm^2^) | Activity (%) |
| **GSLS** |  | |  |  |  |  |  |
| 5 | 339.33±22.05 | | 20.03±1.78d | 92.66±6.00 | 43.69±3.05c | 108.94±45.03 | 24.82±4.33d |
| CK1 | 509.12±22.61 | | -- | 235.58±16.09 | -- | 175.33±59.58 | -- |
| 10 | 243.37±103.96 | | 23.24±4.27d | 69.67±18.45 | 48.03±5.30bc | 96.67±12.58 | 30.67±6.01cd |
| CK2 | 382.67±131.19 | | -- | 205.46±81.50 | -- | 183.33±30.55 | -- |
| 25 | 211.45±63.84 | | 31.83±1.64c | 111.33±15.18 | 53.79±3.27b | 63.67±8.08 | 38.99±1.60bc |
| CK3 | 407.30±115.47 | | -- | 369.85±17.58 | -- | 145.77±17.35 | -- |
| 50 | 130.93±25.53 | | 48.48±2.96b | 49.07±13.23 | 65.5±5.12a | 48.67±17.39 | 42.61±9.21b |
| CK4 | 378.67±99.08 | | -- | 239.16±76.00 | -- | 119.53±35.09 | -- |
| 100 | 95.76 ±54.77 | | 54.07±2.32a | 36.67±6.66 | 72.64±4.86a | 56.33±16.29 | 53.96±3.77a |
| CK5 | 327.68±200.01 | | -- | 231.33±5.69 | -- | 187.66±45.52 | -- |
| **PDS** |  | |  |  |  |  |  |
| 5 | 187.35±18.54 | | 22.62±3.79d | 110.28±16.26 | 22.31±1.02e | 92.167.50 | 14.01±0.81c |
| CK1 | 299.65±28.19 | | -- | 174.15±13.07 | -- | 122.40±10.63 | -- |
| 10 | 215.45±21.59 | | 29.28±1.42c | 105.05±6.08 | 37.29±2.05d | 124.30±10.47 | 19.86±4.07c |
| CK2 | 393.23±22.66 | | -- | 230.75±14.93 | -- | 185.12±12.19 | -- |
| 25 | 185.33±16.92 | | 46.98±1.44b | 89.37±7.29 | 46.35±1.42c | 104.53±6.81 | 28.93±4.01b |
| CK3 | 513.57±28.50 | | -- | 244.32±12.36 | -- | 190.44±13.12 | -- |
| 50 | 97.27±5.80 | | 67.81±2.46a | 54.36±7.05 | 69.02±5.37b | 159.36±17.50 | 34.99±2.41b |
| CK4 | 508.23±31.24 | | -- | 296.65±20.09 | -- | 332.38±22.70 | -- |
| 100 | 84.25±14.00 | | 70.88±2.34a | 23.35±18.08 | 80.9±4.36a | 65.25±5.51 | 47.88±4.71a |
| CK5 | 492.18±9.64 | | -- | 220.55±23.67 | -- | 179.95±11.65 | -- |
| **PTS** |  |  | |  |  |  |  |
| 5 | 140.67±27.59 | 18.57±0.96c | | 114.33±12.01 | 28.62±4.90c | 69.33±8.50 | 28.25±5.62c |
| CK1 | 205.33±44.05 | -- | | 208.33±42.03 | -- | 126.67±28.54 | -- |
| 10 | 288.67±53.11 | 20.37±2.40c | | 174.67±48.06 | 31.77±2.56c | 63.33±23.35 | 29.11±0.49c |
| CK2 | 437.33±85.56 | -- | | 336.35±82.18 | -- | 110.33±39.72 | -- |
| 25 | 232.33±14.29 | 25.27±0.99b | | 122.67±27.02 | 34.76±4.30c | 66.33±11.37 | 31.23±2.51c |
| CK3 | 389.67±27.57 | -- | | 251.25±33.78 | -- | 126.67±22.03 | -- |
| 50 | 102.33±36.12 | 33.23±3.10a | | 110.33±18.01 | 42.33±2.59b | 55.33±10.02 | 35.04±1.91ab |
| CK4 | 205.12±73.30 | -- | | 265.67±47.23 | -- | 115.67±25.17 | -- |
| 100 | 125.67±10.41 | 37.89±4.13a | | 58.67±2.52 | 58.37±3.58a | 74.33±9.02 | 42.18±8.03a |
| CK5 | 280±40.50 | -- | | 225.33±34.67 | -- | 186.67±45.61 | -- |

Data are expressed as mean ± SD. Letters after the data indicate analysis using one-way ANOVA followed by a post hoc Dunnet’s test for comparison.. GSLS, toatal ginsenoside of ginseng stems and leaves; PDS, panaxadiols saponins; PTS, panaxatriol saponins. F. area, feeding leaf area, calculated as: origin area of leaf discs – remaining area (ReA) of leaf disc. Antifeedant activity for choice bioassay was computed as: antifeedant activity (%) = (ReA_control_ - ReA_test_)×100%/(ReA_control_ + ReA_test_)
