# Supplementary material for: Antifeedant and ovicidal activities of ginsenosides against Asian corn borer, Ostrinia furnacalis (Guenee)
Source: PLoS One. 2019 Feb 15;14(2):e0211905. doi: 10.1371/journal.pone.0211905 (PMC6377112; doi:10.1371/journal.pone.0211905)
Supplement: S4 Table — (DOCX) [file pone.0211905.s004.docx]

Supplementary Table S4. No-choice antifeedant activity of different ginsenoside monomers against the 3^rd^-instar larvae of *O. furnacalis*.

| Monomers | Concentration (mg/mL) | Antifeedant activity (%) | | |
| --- | --- | --- | --- | --- |
|  |  | 24 h | 48 h | 72 h |
| Rb1 | 0.25 | 33.47±3.20b | 48.93±1.42c | 43.57±7.17d |
|  | 0.5 | 46.36±6.29a | 51.89±2.53c | 49.06±1.59bc |
|  | 1.0 | 50.18±2.79a | 57.97±2.32b | 52.55±6.25bc |
|  | 2.0 | 49.83±3.40a | 63.40±3.06a | 57.82±7.43ab |
|  | 4.0 | 52.49±6.45a | 66.53±4.31a | 62.17±4.68a |
| Rb2 | 0.25 | 29.38±3.51c | 50.78±4.68c | 40.58±5.86d |
|  | 0.5 | 43.38±16.52ab | 49.34±4.42c | 44.40±4.57d |
|  | 1.0 | 39.63±2.58ab | 59.69±3.39b | 49.76±2.13bc |
|  | 2.0 | 52.79±25.74ab | 62.33±4.12b | 56.01±10.72ab |
|  | 4.0 | 55.13±12.77a | 72.32±5.68a | 63.92±2.40a |
| Rc | 0.25 | 30.86±3.63e | 45.92±8.37d | 42.40±14.63c |
|  | 0.5 | 37.89±7.96cd | 48.72±4.67bc | 43.72±5.63c |
|  | 1.0 | 44.94±1.07bc | 56.17±4.37b | 48.39±10.29c |
|  | 2.0 | 50.37±1.07ab | 65.90±4.29a | 52.23±7.13ab |
|  | 4.0 | 55.15±4.02a | 68.21±5.08a | 66.42±9.34a |
| Rd | 0.25 | 32.46±7.03 c | 45.72±2.30d | 42.21±3.91c |
|  | 0.5 | 32.24±7.17 c | 52.83±10.85bc | 44.22±3.89c |
|  | 1.0 | 48.11±4.21 b | 62.21±2.47ab | 47.04±0.25c |
|  | 2.0 | 53.10±8.45 b | 62.69±13.28ab | 57.39±7.337b |
|  | 4.0 | 66.74±7.42 a | 73.75±6.45a | 65.04±3.13a |
| Re | 0.25 | 22.48±3.85e | 43.23±3.32d | 35.21±2.06c |
|  | 0.5 | 30.89±2.61cd | 48.40±2.30bc | 37.44±3.23c |
|  | 1.0 | 38.47±7.01bc | 50.92±4.06b | 44.71±7.41ab |
|  | 2.0 | 47.47±10.08ab | 53.75±3.96b | 52.74±3.11a |
|  | 4.0 | 50.47±1.71a | 59.55±5.03a | 51.52±12.11a |
| Rg1 | 0.25 | 32.46±7.03b | 43.44±3.96d | 40.75±2.68c |
|  | 0.5 | 32.11±6.11b | 47.31±5.95bc | 41.45±9.23c |
|  | 1.0 | 33.94±2.96b | 53.31±4.42ab | 46.31±3.23ab |
|  | 2.0 | 43.84±2.27a | 59.95±5.22a | 52.11±5.63ab |
|  | 4.0 | 49.56±2.69a | 60.69±7.02a | 53.31±11.22a |

Data are expressed as mean ± SD. Letters after the data indicate analysis using one-way ANOVA followed by a post hoc Dunnet’s test for comparison.. Antifeedant activity for choice bioassay was computed as: antifeedant activity (%) = (ReA_control_ - ReA_test_)×100%/(ReA_control_ + ReA_test_).
